# A systematic review and meta-analysis of the effect of statins on plasma asymmetric dimethylarginine concentrations

**DOI:** 10.1038/srep09902

**Published:** 2015-05-13

**Authors:** Corina Serban, Amirhossein Sahebkar, Sorin Ursoniu, Dimitri P. Mikhailidis, Manfredi Rizzo, Gregory Y.H. Lip, G. Kees Hovingh, John J.P. Kastelein, Leszek Kalinowski, Jacek Rysz, Maciej Banach

**Affiliations:** 1 Department of Functional Sciences, Discipline of Pathophysiology, “Victor Babes” University of Medicine and Pharmacy, Timisoara, Romania; 2 Biotechnology Research Center, Mashhad University of Medical Sciences, Mashhad, Iran; 3 Metabolic Research Centre, Royal Perth Hospital, School of Medicine and Pharmacology, University of Western Australia, Perth, Australia; 4 Department of Functional Sciences, Chair of Public Health, “Victor Babes” University of Medicine and Pharmacy, Timisoara, Romania; 5 Department of Clinical Biochemistry, Royal Free Campus, University College London Medical School, University College London (UCL), London, UK; 6 Biomedical Department of Internal Medicine and Medical Specialties, University of Palermo, Italy; 7 University of Birmingham Centre for Cardiovascular Sciences, City Hospital, Birmingham, UK; 8 Department of Vascular Medicine, Academic Medical Center, Amsterdam, Netherlands; 9 Department of Medical Laboratory Diagnostics, Medical University of Gdansk, Gdansk, Poland; 10 Department of Nephrology, Hypertension and Family Medicine, Chair of Nephrology and Hypertension, Medical University of Lodz, Poland; 11 Department of Hypertension, Chair of Nephrology and Hypertension, Medical University of Lodz, Poland

## Abstract

The impact of statin therapy on plasma asymmetric dimethylarginine (ADMA) levels has not been conclusively studied. Therefore the aim of the meta-analysis was to assess the effect of statins on circulating ADMA levels. We searched selected databases (up to August 2014) to identify randomized controlled trials (RCTs) that investigate the effect of statins on plasma ADMA concentrations. A weighted meta-regression (WMD) using unrestricted maximum likelihood model was performed to assess the impact of statin dose, duration of statin therapy and baseline ADMA concentrations as potential variables on the WMD between statin and placebo group. In total, 1134 participants in 9 selected RCTs were randomized; 568 were allocated to statin treatment and 566 were controls. There was a significant reduction in plasma ADMA concentrations following statin therapy compared with placebo (WMD: − 0.104 μM, 95% confidence interval: − 0.131 to − 0.077, Z = − 7.577, *p* < 0.0001). Subgroups analysis has shown a significant impact of hydrophilic statins (WMD: − 0.207 μM, 95%CI: − 0.427 to + 0.013, Z = − 7.250, *p* < .0001) and a non-significant effect of hydrophobic statins (WMD: − 0.101 μM, 95%CI: − 0.128 to − 0.074, Z = − 1.845, *p* = 0.065). In conclusion, this meta-analysis of available RCTs showed a significant reduction in plasma ADMA concentrations following therapy with hydrophilic statins.

Endothelial dysfunction is an early event in atherogenesis characterized by decreased availability of nitric oxide (NO), which diffuses towards the vascular smooth muscle tissues (VSMCs), triggers a rise of intracellular cyclic guanosine monophosphate (cGMP), leading to vasorelaxation^1^. Endothelial dysfunction may be associated with increased circulating asymmetric dimethylarginine (ADMA) levels - an L-arginine analogue, which inhibits NO formation^2^. ADMA is a pan-inhibitor of all 3 NO synthases (NOS) isoforms (potent noncompetitive inhibitor of neuronal NOS and week inhibitor of inducible and endothelial NOS)^3^ and its plasma concentrations in the general population is 0.4–0.7 μM^4^.

The first study that showed that middle-aged smoking men in the highest quartile of ADMA levels were at an almost 4-fold risk for acute coronary events was conducted in 2001^5^. Since then, it has been shown that higher ADMA levels are related to increased mortality and adverse clinical outcomes in patients with coronary artery disease (CAD), diabetes, renal disease and ischemic stroke^6^,^7^,^8^,^9^. Moreover, in the Coronary Artery Risk Determination investigating the Influence of ADMA Concentration (CARDIAC) study, ADMA was shown to be a risk factor for CAD, independently of traditional predictors^10^.

Formation of NO is regulated by both substrate availability (L-arginine) and the presence of the inhibitor (ADMA), which in turn may be represented by their ratio^11^. However, the application of L-arginine/ADMA ratio is much limitted due to the fact that L-arginine varies much stronger that ADMA levels in the circulation, and therefore the ratio need not reflect the intracellular situation^10^. The Hoorn Study showed that systemic inflammation was associated with decreased arginine and increased ADMA plasma levels resulting in an unfavorable NOS substrate-to-inhibitor ratio^12^.

The interplay of inflammation, endothelial dysfunction, and oxidative stress might play a crucial role in ADMA pathophysiology, and reduction of ADMA levels might be a significant target for preventing endothelial dysfunction^13^. Statins may provide an effective response to reverse endothelial dysfunction *via* reduction of ADMA levels; however, the available evidence is not conclusive. Therefore, the aim of this systematic review and meta-analysis was to assess the impact of statins on circulating ADMA levels.

## Methods

### Data Sources

This study was designed in conformity to the guidelines of the 2009 Preferred Reporting Items for Systematic Reviews and Meta-Analysis (PRISMA) statement^14^. Our search included PubMed, Web of Science, Cochrane Library, Scopus and EMBASE databases and was limited to randomized controlled trials (RCTs) carried out from January 1, 1970 to August 1, 2014, investigating the potential effects of statins on circulating ADMA levels. The references of relevant publications were searched and articles of interest were retrieved. The databases were searched using the following search terms in titles and abstracts (also in combination with MESH terms): (rosuvastatin OR pravastatin OR fluvastatin OR simvastatin OR atorvastatin OR pitavastatin OR lovastatin OR cerivastatin OR “statin therapy” OR statins) AND (ADMA OR “asymmetric dimethylarginine”). The wild-card term ‘‘*’’ was used to increase the sensitivity of the search strategy. Two reviewers (CS and AS) evaluated each article separately. Disagreements were resolved by agreement and discussion with a third party (MB). Uncontrolled studies or those with results that did not consider the main objectives of the meta-analysis were omitted.

### Study selection

#### Inclusion criteria

Study design had to meet the following criteria: (1) randomized, placebo-controlled parallel or cross-over trial, (2) population enrolled: adults ≥ 18 years, and, (3) plasma ADMA levels at baseline and after statin administration were available.

#### Exclusion criteria

The studies were excluded if: (1) had a non-randomized or uncontrolled design, (2) the study was not conducted in statin-treated subjects, (3) no numerical values were presented concerning plasma ADMA levels at baseline and at the end of the study, (3) had duplicate data on ADMA concentrations, (4) we were unable to obtain adequate details of study methodology or results from the article or the investigators, and, (5) the study was an ongoing trial.

#### Quality assessment

The quality of involved studies in this meta-analysis was evaluated using Jadad scale^15^. This scale includes randomization (0–2 points), blinding (0–2 points), and dropouts and withdrawals (0–1 point). The overall score of a study in accordance with this scale varies among 0-5, with greater scores as a measure of better quality^16^. Studies with Jadad scale of ≤ 2 and ≥ 3 were considered as low- and high-quality, respectively^17^.

#### Quantitative Data Synthesis

Meta-analysis was conducted using Comprehensive Meta-Analysis (CMA) V2 software (Biostat, NJ). Since all studies used the same methods for the measurement of ADMA levels (plasma levels measured in μM), weighted raw mean difference and 95% confidence interval (CI) was used as summary statistic. Weighting of results was performed using the inverse variance method (Borenstein M, *et al.* Comprehensive meta-analysis version 2. Engelwood, NJ: Biostat, 2005). Mean difference in measurements was calculated as follows: (measure at end of follow-up in the statin group − measure at baseline in the statin group) − (measure at end of follow-up in the placebo group − measure at baseline in the placebo group). Standard deviations (SDs) of the mean difference were calculated using the following formula: SD = square root [(SD_pre-treatment_)^2^ + (SD_post-treatment_)^2^ − (2R × SD_pre-treatment_ × SD_post-treatment_)], assuming a correlation coefficient (R) = 0.5^18^. A random-effect model and the generic inverse variance method were used for quantitative data synthesis in order to address the inter-study variations in time of statin type, statin dose and duration of treatment. Pooled effect size was expressed as weighted mean difference (WMD) with 95%CI. In order to evaluate the influence of each study on the overall effect size, sensitivity analysis was conducted using the one-study remove (leave-one-out) approach^19^,^20^. In case the values were only presented as graph, the software GetData Graph Digitizer 2.24 (http://getdata-graph-digitizer.com/) was applied to digitize and extract the data; otherwise the authors of the article were contacted to provide numerical values of ADMA concentrations in statin and/or placebo group.

A weighted meta-regression using unrestricted maximum likelihood model was performed to assess the impact of statin dose, duration of statin therapy and baseline ADMA concentrations as potential moderator variables on the WMD in ADMA concentrations between statin and placebo group^19^,^21^.

Presence of publication bias was explored graphically using funnel plots of precision (1/standard error) by study effect size (mean difference). Asymmetric funnel plot was further assessed for publication bias using Duval & Tweedie trim-and-fill and classic “fail-safe N” methods, as well as Begg’s rank correlation and Egger’s weighted regression tests^18^,^19^.

## Results

### Search results and trial flow

**Figure 1:**
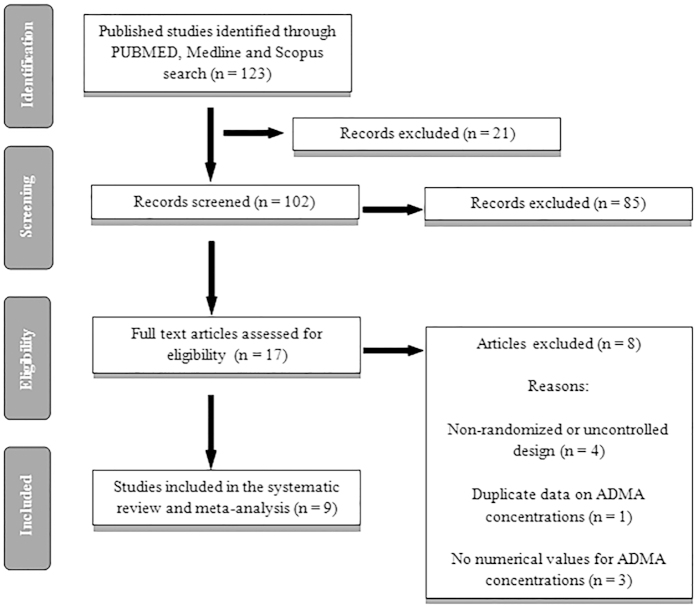
Flow chart of number of studies identified and included into the meta-analysis.

A summary of the study selection process is shown in Fig. 1. The initial screening for potential relevance excluded articles whose titles and/or abstracts were clearly irrelevant. After removing the trials not assessing the effects of statins in reducing plasma ADMA concentrations, only 17 RCTs met the inclusion criteria and the full-texts were obtained. After assessment 9 articles met the inclusion criteria and were selected for the final meta-analysis.

### Description of studies

In total, 1134 participants in the 9 selected RCTs were randomized; 568 were allocated to statin treatment and 566 were controls. The number of participants in these trials ranged from 53 to 650. The included studies were published between 2003 and 2012, and were conducted in Norway, Taiwan, the Netherlands, Bulgaria, Italy Turkey, New Zealand, China and Finland^22^,^23^,^24^,^25^,^26^,^27^,^28^,^29^,^30^. Statins (pravastatin, rosuvastatin, simvastatin, fluvastatin, and atorvastatin) were administered at doses from 10 to 80 mg/day. Duration of trials ranged between 6 weeks and 24 months. Six trials were designed as parallel-group studies^22,25,27,29-31^ and 2 trials as cross-over studies^23^,^28^. One study^26^ was a prospective follow-up trial conducted in 3 stages. Demographic and baseline parameters of the included studies are shown in Table 1.

### Quantitative data synthesis

**Figure 2:**
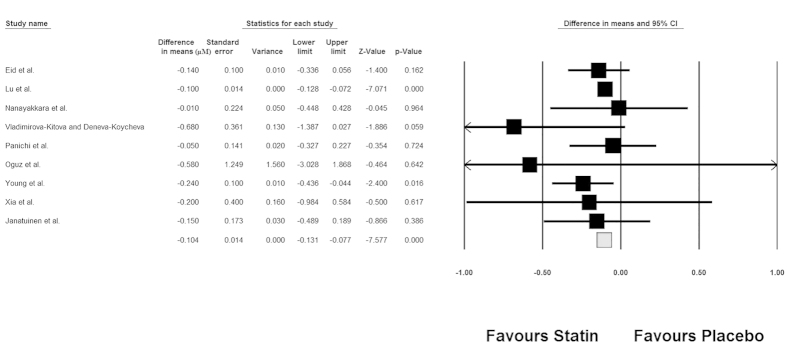
Forest plot detailing weighted mean difference and 95% confidence intervals for the impact of statin therapy on plasma concentrations of ADMA. Meta-analysis was performed using a random-effect model with inverse variance weighting.

**Figure 3:**
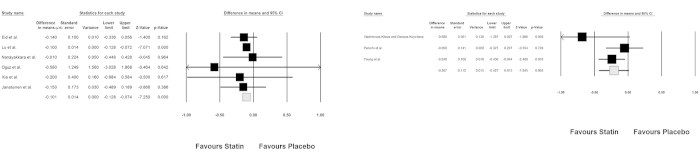
Forest plot detailing weighted mean difference and 95%Cl for the impact of hydrophilic (left) and hydrophobic (right) statins on plasma concentrations of ADMA. Meta-analysis was performed using a random-effect model with inverse variance weighting.

Combining results of retrieved RCTs indicated a significant reduction in plasma ADMA concentrations following treatment with statins compared with placebo (WMD: − 0.104 μM, 95%CI: − 0.131 to − 0.077, Z = − 7.577, *p* < 0.0001). Forest plots detailing the meta-analysis of RCTs assessing the impact of statin therapy on plasma ADMA levels is illustrated in Fig. 2. Subgroup analysis revealed a significant impact of hydrophilic statins (rosuvastatin, pravastatin and fluvastatin; *n* = 403; WMD: − 0.207 μM, 95%CI: − 0.427 to + 0.013, Z = − 7.250, *p* < 0.0001), and a non-significant effect of hydrophobic statins (simvastatin and atorvastatin; *n* = 321; WMD: − 0.101 μM, 95%CI: − 0.128 to − 0.074, Z = − 1.845, *p* = 0.065) (Fig. 3).

**Figure 4:**
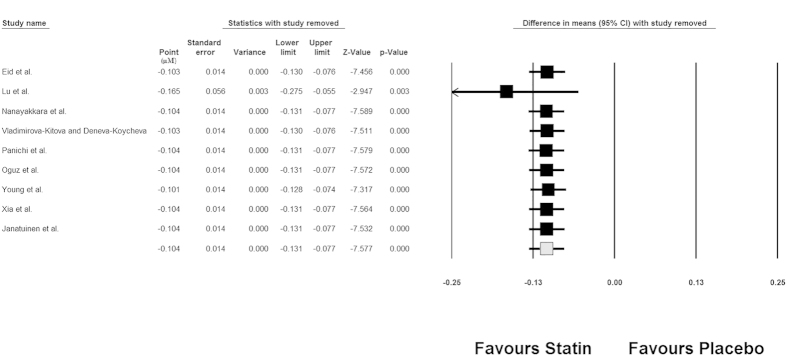
Leave-one-out sensitivity analysis for the impact of statin therapy on plasma concentrations of ADMA.

The strength of the pooled estimate was robust and did not significantly differ according to the characteristics of individual studies in the leave-one-out sensitivity analysis. The results of sensitivity analysis are summarized in Fig. 4.

### Meta-regression

Weighted unrestricted maximum likelihood meta-regression analysis was performed to assess the impact of potential moderators on the pooled effect size. None of the moderator parameters i.e. statin dose (slope: − 0.003; 95%CI: − 0.006 to 0.001; *p* = 0.164), duration of statin therapy (slope: 0.001; 95%CI: − 0.008 to 0.011; *p* = 0.774) and baseline ADMA concentrations (slope:  − 0.083; − 0.275 to 0.109; *p* = 0.399) was significantly associated with the pooled estimate of the statin effect on plasma ADMA concentrations. Meta-regression bubble plots are illustrated in Supplementary Figure S1.

### Publication bias

Visual inspection of funnel plot asymmetry suggested potential publication bias for the effects of statin therapy on plasma ADMA concentrations (Supplementary Figure S2). Imputation of theoretically missed studies using Duval and Tweedie’s trim-and-fill method added 3 studies, leading to an imputed WMD of − 0.100 (95%CI: − 0.127 to − 0.074) which was still significant (Supplementary Figure S2). The “fail safe N” test showed that 50 theoretically missing studies would be needed to add to the analysis of the effect of statin therapy on plasma ADMA concentrations in order to yield a statistically non − significant overall effect. Likewise, Begg’s rank correlation test (Kendall’s Tau with continuity correction = − 0.167, Z = 0.626, two-tailed *p* = 0.532) and Egger’s linear regression tests suggested no evidence of publication bias (intercept = − 0.478, 95%CI = − 1.150 to 0.195, *t* = 1.679, df = 7.00, two-tailed *p* = 0.137).

## Discussion

To our knowledge this meta-analysis is the first that assessed the effects of statin therapy on plasma levels of ADMA. The findings provide a thorough synthesis of results from available RCTs and showed a significant reduction in plasma ADMA concentrations. Additionally, statin therapy was examined by class: hydrophobic (simvastatin and atorvastatin), that might be dispersed at low levels throughout human tissues and hydrophilic (pravastatin, rosuvastatin and fluvastatin) that functions mainly in the liver and are present in the circulation^31^,^32^. In our meta-analysis hydrophilic statins (rosuvastatin, pravastatin and fluvastatin) had a significant impact on ADMA levels while hydrophobic statins (simvastatin and atorvastatin) non-significantly reduced ADMA levels.

Currently ADMA is considered a prognostic marker of cardiovascular disease and mortality^6^. The available data also suggests that ADMA has been involved in systemic vascular inflammation through induction of reactive oxygen species (ROS) in endothelial cells^32^. In patients undergoing coronary bypass surgery, it was observed that ADMA levels were correlated with elevated NOS-derived generation of ROS^33^. Furthermore, it has been shown that ROS upregulate ADMA synthesis and protein arginine N-methyltransferase expression^34^. In cell culture studies, it has been shown that pro-oxidant and pro-inflammatory stimulants inhibit dimethylarginine dimethylaminohydrolase (DDAH) activity^35^. Decreased DDAH, the enzyme responsible for ADMA degeneration, is generally followed by the consecutive decrease of NOS activity, increase of ADMA concentrations and development of atherosclerosis^36^,^37^. However, it should also be mentioned that there are some doubts on the ADMA/DDAH association – e.g. DDAH activity is not associated with oxidative stress in the elderly patients with peripheral arterial occlusive disease^38^,^39^. In human monocytic cells, ADMA induces tumor necrosis factor (TNF)-α production *via* the inhibitory effect of reinioside C and ROS/nuclear factor (NF)–κB dependent pathways^40^. Since both ROS and systemic inflammation are responsible for increased ADMA levels, and statins are recognized as anti-inflammatory and antioxidant agents^41^, the hypothesis was that statin therapy might decrease ADMA levels. Indeed, several smaller studies have shown that statin therapy reduces ADMA levels 25,42, however other studies with high dose statins (e.g. simvastatin 80 mg/day or atorvastatin 40 mg/day) did not decrease plasma ADMA levels^43^. It seems that our meta-analysis provides the answer to the question on the role of statins on ADMA levels (mainly hydrophilic), irrespective of the statins doses and therapy duration. These results also show the marginal or lack of effect of simvastatin and atorvastatin (hydrophobic statins) on plasma ADMA levels^43^.

There are few hypotheses on how statins influence ADMA levels. One of them concerns the inhibition of ADMA-induced inflammatory reaction, modulated by mitogen-activated protein kinase (MAPK) pathway in human endothelial cells^44^. Statins also activate the transcription factor sterol response element binding protein (SREBP) through decreasing content of the cholesterol in the membrane^45^. SREBP specifically enhances the expression of more than 30 genes associated with the synthesis and uptake of fatty acids, phospholipids, cholesterol and triglycerides^46^. One of its isoforms - nuclear SREBP-2 increases the transcription of proprotein convertase subtilisin/kexin type 9 (PCSK9)^47^. It has been shown that statins upregulate both PCSK9 mRNA levels and LDLR *via* activation of sterol-mediated SREBP-2, an important activator of DDAH transcription and activity^48^. Since reduced DDAH activity is linked to endothelial dysfunction, we speculate that statin therapy might decrease ADMA levels through multiple mechanisms such as activation of sterol-mediated SREBP-2, increasing of transcription of PCSK9 or by decreasing ADMA-induced inflammatory reaction, modulated by MAPK^49^,^50^.

This meta-analysis has several limitations. Most importantly, the eligible RCTs usually had small populations and short follow-up (up to 6 months in 8/9 included studies). The included studies were also heterogeneous with regards to population characteristics (there were patients with hyperlipidemia, renal failure or atrial fibrillation), study design, and statin preparation and dose. In order to cover these variabilities we used a more conservative random-effects model and performed the sensitivity analysis. The meta-regression analysis also revealed that none of the moderator parameters i.e. statin dose, duration of statin therapy and baseline ADMA concentrations were significantly associated with the pooled estimate of statin effect on plasma ADMA concentrations. Finally, the smoking status, an important determinant of ADMA levels (as well as other variables, such as: hyperhomocysteinemia, hypertension, coronary artery disease, heart failure, and administration of the following drugs: antioxidants, estrogen, vitamin A, angiotensin converting enzyme inhibitors, angiotensin AT1 receptor antagonists, and beta-adrenoreceptor blocking drugs), could not be considered in this meta-analysis due to lack of data.

In conclusion, this meta-analysis of RCTs showed a significant reduction in plasma ADMA concentrations following hydrophilic statin therapy. These results might reveal an additional benefit of statins, which might contribute to the observed reduction of cardiovascular risk. Larger, well-designed studies involving smoking status are needed to validate our findings.

## Author Contributions

CS - designed the study, made the literature search, drafted the manuscript, prepared the revised version; AS - designed the study, made the statistical analysis, corrected the draft of the paper; SU - made the statistical analysis, drafted the manuscript; DPM, MR, GYHL, GKH, JJPK, LK, JR - corrected the draft of the paper and the revised version; MB - designed the study, made the literature search, drafted the manuscript, prepared the revised version, submitted the paper.

## Additional Information

**How to cite this article**: Serban, C. *et al.* A systematic review and meta-analysis of the effect of statins on plasma asymmetric dimethylarginine concentrations *Sci. Rep.* **5**, 09902; doi: 10.1038/srep09902 (2015).

## Supplementary Material

Supporting Information Supplementary Figures

## Figures and Tables

**Table 1:** Demographic characteristics of the included studies.

		**Panichi** ***et al.*****[22]**	**Eid** ***et al.*** **[23]**	**Lu** ***et al.*****[24]**	**Nanayakkara** ***et al.*****[25]**	**Vladimira-Kitova** ***et al*****[26]**	**Oguz** ***et al.*****[27]**	**Young** ***et al.*****[28]**	**Xia** ***et al.*** **[29]**	**Janatuinen** ***et al.*** **[30]**	
**Year**		2008	2003	2004	2009	2012	2008	2008	2009	2003	
**Jadad score**		3	4	3	3	3	3	3	4	3	
**Location**		Italy	Norway	Taiwan	Netherlands	Bulgaria	Turkey	New Zealand	China	Finland	
**Design**		Randomized double-blinded placebo-controlled parallel trial	Double blinded, placebo-controlled cross-over trial	Multicenter, randomized, double-blinded, placebo-controlled parallel trial	Secondary analysis of a randomized double-blind placebo-controlled parallel trial	Prospective follow-up randomized controlled trial conducted in three stages	Randomized controlled parallel trial	Randomized double-blinded placebo-controlled cross-over trial	Randomized controlled parallel trial	Randomized double-blinded placebo-controlled parallel trial	
**Duration of trial**		6 months	8 weeks	6 weeks	24 months	3 months	6 weeks	6 weeks	3 months	6 months	
**Inclusion criteria**		Patients with chronic kidney diseases (creatinine clearance ranging from 15 to 60 ml/min/1.73 m^2^) and LDL cholesterol > 100 mg/dL	Men with untreated hypercholesterolemia	Patients with hypercholesterolemia with fasting plasma LDL cholesterol > 160 mg/dl and triglyceride levels < 350 mg/dl after an initial 6 weeks of diet control	Patients with creatinine clearance of 15 to 70 mL/min/1.73 m^2^ (according to the Cockcroft-Gault equation)	Patients over 16 years of age with severe hypercholesterolemia defined as fasting total cholesterol level ≥ 7.5 mmol/l and LDL-C level of ≥ 4.9 mmol/l and a family history of premature atherosclerosis.	Patients over 20 years of age with diagnosis of metabolic syndrome, a LDL cholesterol level between 100-160 mg/dL, and a triglyceride level lower than 400 mg/dL.	Patients with symptomatic heart failure (ejection fraction < 40%, New York Heart Association Functional Classes II and III)	Patients consecutively subjected to elective electrical cardioversion to treat persistent atrial fibrillation ( > 48 h).	Men aged 25–40 years; total cholesterol levels 5.5–9.0 mmol/l measured previously at routine controls provided by employers; otherwise healthy; no continuous medication or use of antioxidant vitamins.	
**Statin intervention**		Simvastatin 40 mg/day	Pravastatin 40 mg/day	Rosuvastatin 10 mg/day	Pravastatin 40 mg/day	Simvastatin 40 mg/day	Simvastatin 80 mg/day	Fluvastatin 80 mg/day	Atorvastatin 40 mg/day	Rosuvastatin 10 mg/day	Pravastatin 40 mg/day
**Participants**	Treatment	20	32	23	46	325	120	42	23	32	25
	Control	15	32	23	47	325	120	43	23	32	26
**Age (years)**	Treatment	60 ± 12	33-71	62.8 ± 11.2	54 ± 11	46 ± 4	46 ± 3	55.50 ± 10.46	60.7 ± 10.4	62.28 ± 8.55	35.7 ± 3.6
	Control	58 ± 11	33-71	59.8 ± 11.8	52 ± 13	46 ± 2	46 ± 2	56.16 ± 7.56	60.7 ± 10.4	60.72 ± 8.21	34.6 ± 4.3
**Male (%)**	Treatment	70.0	100.0	43.5	52.1	51.1	46.7	38.1	NS	68.7	100.0
	Control	60.0	100.0	73.9	61.7	52.3	49.2	37.2	NS	62.5	100.0
**BMI (kg/m**^**2**^)	Treatment	25.1 ± 3.0	NS	25.3 ± 2.7	27 ± 5	25 ± 2	24 ± 4	NS	NS	23.74 ± 2.26	25.3 ± 2.8
	Control	24.9 ± 2.3	NS	24.8 ± 2.9	26 ± 4	25 ± 3	25 ± 2	NS	NS	23.49 ± 2.20	24.6 ± 1.8
**Baseline plasma ADMA concentration (μM)**	Treatment	0.90 ± 0.10	1.50 (1.18, 1.75)*	0.60 ± 0.19	0.53 ± 0.06	1.17 ± 0.15	1.26 ± 0.38	1.57 ± 1.07	NS	1.60 ± 0.41	0.38 ± 0.18
	Control	0.74 ± 0.12	1.64 (1.24, 1.75)*	0.54 ± 0.14	0.53 ± 0.09	1.16 ± 0.17	1.25 ± 0.21	1.17 ± 1.41	NS	1.58 ± 0.40	0.42 ± 0.15

Values are expressed as mean ± SD. ^*^Median values and 25, 75 percentiles are given;

ABBREVIATIONS: BMI: body mass index; LDL-C: low-density lipoprotein cholesterol; NA: not applicable, NS: not stated.
