# Supplementary material for: A systematic review and meta-analysis of the effect of statins on plasma asymmetric dimethylarginine concentrations
Source: Sci Rep. 2015 May 13;5:9902. doi: 10.1038/srep09902 (PMC4429557; doi:10.1038/srep09902)

A SYSTEMATIC REVIEW AND META-ANALYSIS OF THE EFFECT OF STATINS ON PLASMA ASYMMETRIC DIMETHYLARGININE CONCENTRATIONS

Corina Serban[1#], Amirhossein Sahebkar[2,3#], Sorin Ursoniu[4], Dimitri P. Mikhailidis[5], Manfredi Rizzo[6], Gregory Y.H. Lip[7], G. Kees Hovingh[8], John J.P. Kastelein[8]; Leszek Kalinowski[9], Jacek Rysz[10], Maciej Banach[11]; *Lipid and Blood Pressure Meta-analysis Collaboration (LBPMC) Group*

#*These authors contributed equally to this work.*

[1]Department of Functional Sciences, Discipline of Pathophysiology, "Victor Babes" University of Medicine and Pharmacy, Timisoara, Romania; [2]Biotechnology Research Center, Mashhad University of Medical Sciences, Mashhad, Iran; [3]Metabolic Research Centre, Royal Perth Hospital, School of Medicine and Pharmacology, University of Western Australia, Perth, Australia; [4]Department of Functional Sciences, Chair of Public Health, "Victor Babes" University of Medicine and Pharmacy, Timisoara, Romania; [5]Department of Clinical Biochemistry, Royal Free Campus, University College London Medical School, University College London (UCL), London, UK; [6]Biomedical Department of Internal Medicine and Medical Specialties, University of Palermo, Italy; [7]University of Birmingham Centre for Cardiovascular Sciences, City Hospital, Birmingham, UK; [8]Department of Vascular Medicine, Academic Medical Center, Amsterdam, Netherlands; [9]Department of Medical Laboratory Diagnostics, Medical University of Gdansk, Gdansk, Poland; [10]Department of Nephrology, Hypertension and Family Medicine, Chair of Nephrology and Hypertension, Medical University of Lodz, Poland; [11]Department of Hypertension, Chair of Nephrology and Hypertension, Medical University of Lodz, Poland.

SUPPLEMENTARY FIGURES LEGENDS:

Supplementary Figure S1. Weighted unrestricted maximum likelihood meta-regression analysis of the impact of statin dose (A), duration of statin therapy (B), and baseline ADMA concentrations (C) on the pooled effect size. The size of each circle is inversely proportional to the variance of change.

Supplementary Figure S2. Funnel plot detailing publication bias in the studies reporting the impact of statin therapy on plasma concentrations of ADMA. Open circles represent observed published studies; closed circles represent imputed unpublished studies.

Supplemental figure S1

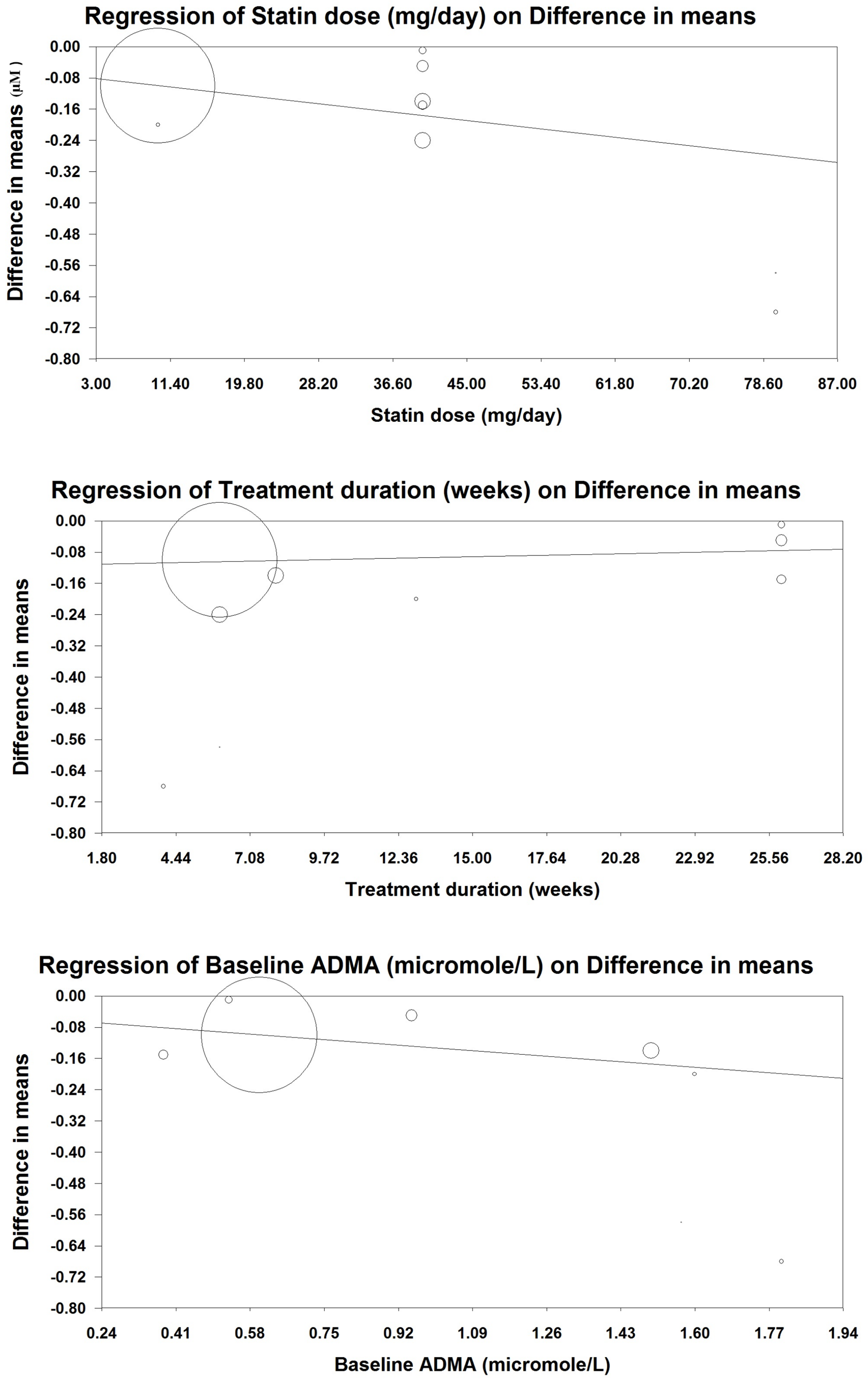

Supplemental figure S2

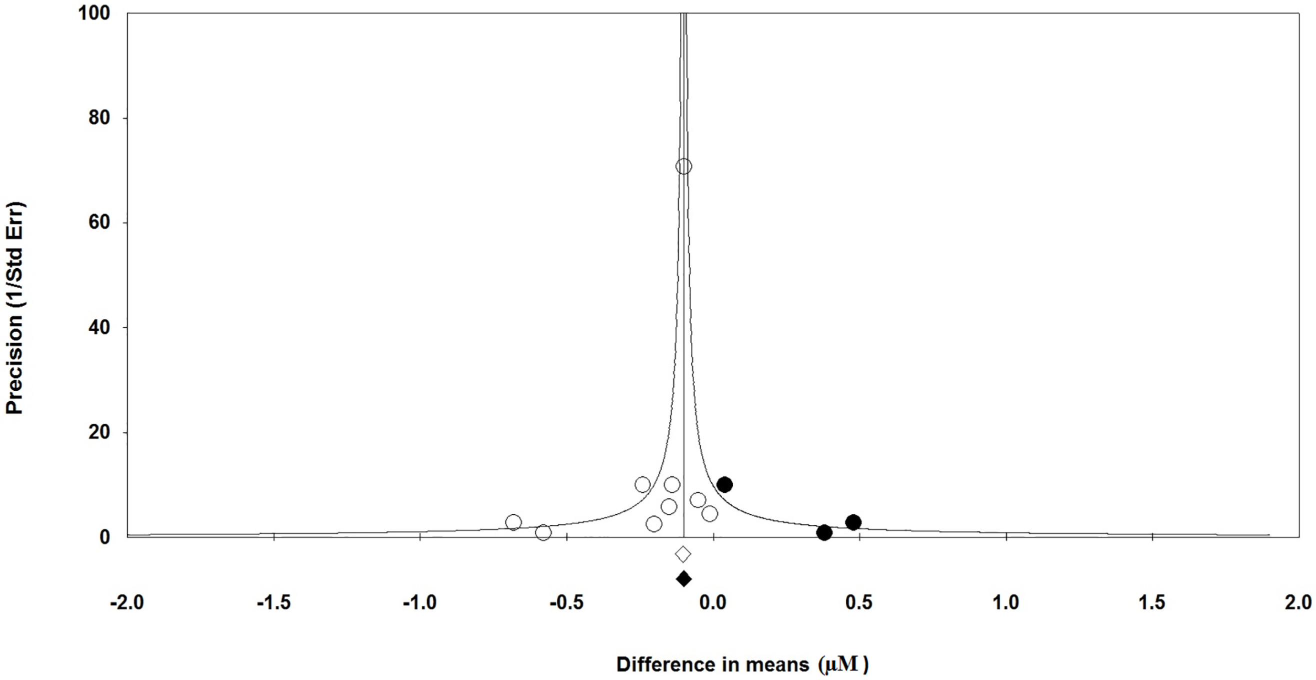

Supplement: Supporting Information — Supplementary Figures [file srep09902-s1.pdf]
